# Key Role of Drug Shops and Pharmacies for Family Planning in Urban Nigeria and Kenya

**DOI:** 10.9745/GHSP-D-16-00197

**Published:** 2016-12-23

**Authors:** Meghan Corroon, Essete Kebede, Gean Spektor, Ilene Speizer

**Affiliations:** a Carolina Population Center, University of North Carolina at Chapel Hill, Chapel Hill, NC, USA.; b Carolina Population Center, University of North Carolina at Chapel Hill, Chapel Hill, NC, USA. Now with North Carolina Department of Health and Human Services, Division of Public Health, Raleigh, NC, USA.; c University of North Carolina at Chapel Hill, Gillings School of Global Public Health, Chapel Hill, NC, USA.

## Abstract

**Background:**

The Family Planning 2020 initiative aims to reach 120 million new family planning users by 2020. Drug shops and pharmacies are important private-sector sources of contraception in many contexts but are less well understood than public-sector sources, especially in urban environments. This article explores the role that drug shops and pharmacies play in the provision of contraceptive methods in selected urban areas of Nigeria and Kenya as well as factors associated with women's choice of where to obtain these methods.

**Methods:**

Using data collected in 2010/2011 from representative samples of women in selected urban areas of Nigeria and Kenya as well as a census of pharmacies and drug shops audited in 2011, we examine the role of drug shops and pharmacies in the provision of short-acting contraceptive methods and factors associated with a women's choice of family planning source.

**Results:**

In urban Nigeria and Kenya, drug shops and pharmacies were the major source for the family planning methods of oral contraceptive pills, emergency contraceptives, and condoms. The majority of injectable users obtained their method from public facilities in both countries, but 14% of women in Nigeria and 6% in Kenya obtained injectables from drug shops or pharmacies. Harder-to-reach populations were the most likely to choose these outlets to obtain their short-acting methods. For example, among users of these methods in Nigeria, younger women (<25 years old) were significantly more likely to obtain their method from a drug shop or pharmacy than another type of facility. In both countries, family planning users who had never been married were significantly more likely than married users to obtain these methods from a drug shop or a pharmacy than from a public-sector health facility. Low levels of family planning-related training (57% of providers in Kenya and 41% in Nigeria had received training) and lack of family planning promotional activities in pharmacies and drug shops in both countries indicate the need for additional support from family planning programs to leverage this important access point.

**Conclusions:**

Drug shops and pharmacies offer an important and under-leveraged mechanism for expanding family planning access to women in urban Nigeria and Kenya, and potentially elsewhere. Vulnerable and harder-to-reach groups such as younger, unmarried women and women who do not yet have children are the most likely to benefit from increased access to family planning at drug shops and pharmacies.

## Abstract (precis)

Pharmacies and drug shops provide a rich opportunity for expanding family planning access to urban women, especially unmarried and younger women. In urban Nigeria and Kenya, drug shops and pharmacies were the major sources for most short-acting methods, including oral contraceptive pills, emergency contraceptives, and condoms.

## INTRODUCTION

The Family Planning 2020 (FP2020) initiative aims to expand access to family planning information, services, and supplies with the ambitious goal of increasing the number of new family planning users by 120 million women and girls by 2020.^1^ This initiative builds upon the wider ongoing global movement toward reproductive rights and universal access to sexual and reproductive health services, as also seen in the recent Sustainable Development Goals.^2^

As focus countries of the FP2020 initiative, Nigeria and Kenya made commitments to improve access to family planning at the London Summit on Family Planning in 2012. Specifically, the government of Nigeria committed to increasing the national contraceptive prevalence rate to 36% by 2018.^3^ In 2013, 10% of currently married women ages 15–49 were using modern contraceptive methods, representing an increase of nearly 6 percentage points from 1990 and 2 percentage points from 2003.^4^^,^^5^ Unmet need for family planning—that is, the percentage of married women who report that they want to delay or limit childbearing but are not using a family planning method—was 16% in 2013.^4^ While this unmet need level combined with low CPR suggests low overall demand for family planning, with a projected growth in population to almost 400 million people by 2050, the family planning needs become immense.^6^

The Government of Kenya committed to increasing the contraceptive prevalence rate from 46% in 2009 to 56% in 2015 and 70% in 2030. In 2014, 53% of currently married women ages 15–49 were using modern contraceptive methods; this is an increase of 21 percentage points from 2003 and 14 percentage points from 2008–09.^7^^,^^8^ Unmet need was 18% in 2014, suggesting continued demand for family planning even in the context of higher use.

In both Nigeria and Kenya in the most recent Demographic and Health Surveys, most of the method use is comprised of shorter-acting methods, such as injectable contraceptives, condoms, and oral contraceptive pills, rather than long-acting reversible contraceptives (LARCs) or sterilization. In Nigeria, about 86% of current contraceptive users ages 15–49 are using one of the following short-acting modern methods: male condoms (44%), injectables (24%), or oral contraceptive pills (18%).^4^ Likewise in Kenya, 80% of women ages 15–49 using a modern method are using either injectables (54%), pills (17%), or male condoms (9%).^8^ These methods are often available outside formal medical facilities and expansion of access to these methods by the non-medical sector could lead to significant increases in method use.^9^

Expanding access to contraceptive methods through the private sector and community-based sources—which includes task sharing (or task shifting)—is an important strategy to help achieve national FP2020 goals and, in particular, aims to reduce barriers to access for youth and lower-income and other marginalized groups of potential family planning users.^10^ Pharmacies and drug shops (also known as chemists or proprietary patent medicine vendors [PPMVs] in Nigeria) are often preferred by young, single, and other underserved populations.^11^^,^^12^ Shifting contraceptive services across different types of supply outlets can increase access for potential family planning users.^13^ Pharmacies and drug shops offer easy access to a number of common contraceptive methods including condoms, oral contraceptive pills, and emergency contraception. Women are able to purchase injectable contraceptives in some contexts in the private non-medical sector, but they are often not able to obtain the injection at the same site; however, regulations are beginning to change to expand access.^9^^,^^14^

Pharmacies and drug shops offer easy access to a number of common contraceptive methods including condoms, pills, and emergency contraception.

Drug shops are privately owned outlets that sell nonprescription drugs. While the legal criteria of drug shops vary by country, usually they are permitted to sell prepackaged patent drugs that are considered to be safe for public use.^12^^,^^14^^,^^15^^,^^16^ Notably, they are generally not allowed to dispense drugs outside of their manufacturer package unlike pharmacies.^9^^,^^12^^,^^16^ In the context of Nigeria, pharmacies and drug shops are distinct. Kenyan law recognizes only pharmacies as a component of the private health sector; however, unregistered pharmacies, also called chemists or drug shops, are widespread.^16^^,^^17^ These drug shops are not in the Kenyan legal structure and thus are not overseen by government regulatory agencies.

Pharmacies in sub-Saharan Africa are usually larger in size than drug shops, carry a wider array of products, and are operated by licensed pharmacists who are authorized to sell both prescription and non-prescription medicines.^18^ Compared with pharmacies, drug shops frequently operate with staff with lower levels of training.^18^ Shortages of trained manpower have limited the number of pharmacies in sub-Saharan Africa; consequently, many countries permit licensed drug shops to offer a limited range of medicines to increase access to more medicines.^18^ In general, drug shops and pharmacies are convenient because they are usually neighborhood-based; sometimes more affordable compared with other private facilities; have the ability to provide services rapidly; operate long hours including weekends; and sometimes offer services in a more confidential manner than other types of sources.^9^^,^^12^^,^^15^

In many cases, drug shops and pharmacies are more accessible to marginalized groups including rural populations and the urban poor.^9^^,^^11^^,^^12^^,^^15^ A study among sexually active women in rural and urban Nigeria showed that younger respondents, single people, Catholics, and Muslims preferred to obtain contraceptives from drug shops than other sources; by contrast, older groups and married respondents made use of hospitals.^10^ A study among clients who purchased emergency contraceptives from private pharmacies in urban areas of Kenya shows that about three-fourths were between ages 20 and 29.^17^ These results are expected given that unmarried women tend to use short-acting methods such as oral contraceptives and condoms, and drug shops and pharmacies are important access points for these methods.^12^^,^^15^ Conversely, married women may be more likely to use long-acting methods, which are predominately available in clinics and hospitals. Moreover, in societies where premarital sex is strongly discouraged, adolescents and unmarried individuals are less likely to get services at conventional health facilities. In addition to provision of contraceptive commodities, evidence shows that drug shops provide family planning information and counseling to their clients.^11^^,^^12^^,^^14^^,^^15^^,^^16^ An observational study on interactions between drug shops and customers in urban and rural Nigeria showed that 25% of the clients interviewed saw drug shops as a source of advice.^16^

Drug shops and pharmacies are often more accessible to marginalized groups.

In this article, we examine the role of the private sector (drug shops and pharmacies) in the provision of family planning methods in selected urban areas of Nigeria and Kenya. We examine factors predictive of using a short-acting method and factors related to where women choose to obtain their method. We also incorporate facility-level data to examine the characteristics of drug shops and pharmacies in these urban settings. A better understanding of private-sector user characteristics along with a more detailed description of private-sector sources of contraceptives can inform programs and strategies to improve access to family planning services in complex urban environments.

## METHODS

The Urban Reproductive Health Initiative, funded by the Bill & Melinda Gates Foundation, aimed to increase modern contraceptive use in selected urban areas of India, Kenya, Nigeria, and Senegal from 2009 to 2014. The initiative in Kenya, known as Tupange, undertook activities in 5 cities: Kakamega, Kisumu, Machakos, Mombasa, and Nairobi. In Nigeria, the initiative was known as the Nigerian Urban Reproductive Health Initiative, which worked in 6 cities: Abuja, Benin City, Ibadan, Ilorin, Kaduna, and Zaria.

The Measurement, Learning & Evaluation (MLE) project was the evaluation component of the Urban Reproductive Health Initiative. Led by the Carolina Population Center at the University of North Carolina (UNC) at Chapel Hill, MLE promoted evidence-based decision making for the initiative through rigorous evaluation methods including a series of longitudinal surveys conducted at baseline, mid-term, and endline. MLE received Institutional Review Board (IRB) approval to conduct the Kenya surveys by the Kenya Medical Research Institute as well as the UNC IRB. MLE received approval to conduct the Nigerian surveys from the Nigeria Ministry of Health Ethics Review Board and the UNC IRB.

### Data and Measures

The MLE baseline surveys collected data on women and men of reproductive age as well as health facilities, health service providers, reproductive health clients, pharmacies, and drug shops. This study draws on data from women, men, pharmacies, and drug shops, collected as part of the MLE baseline surveys in Nigeria and Kenya in 2010/2011.

Both the Nigeria and Kenya women's baseline surveys used a 2-stage cluster sampling approach to select a representative sample of households and women of reproductive age from each project city. In the first stage, a random sample of clusters (or primary sampling units) was selected for each city based on probability proportional to size of the population. In the second stage, a random sample of households was selected in each cluster (41 and 30 households per cluster in Nigeria and Kenya, respectively). All women ages 15–49 in the selected households were eligible for the interview. In Nigeria, a total of 16,144 women successfully completed an interview across the 6 cities between October 2010 and March 2011. In Kenya, a total of 8,932 women completed the interviews across the 5 cities between August 2011 and October 2011. Because of our interest in examining women's use of short-acting methods and the sources of these methods, only women who had sex in the last year in Nigeria and Kenya were included in this analysis. The final weighted sample size of women who had sex in the last year in Nigeria was 11,930, and in Kenya 7,085.

The Nigeria PPMV and pharmacy data were collected between February 2011 and June 2011 from a total of 433 pharmacies and 555 PPMVs. A simple random sampling procedure was used to draw 100 pharmacies per city from an updated master list of pharmacies. However, this target was met only in Abuja and Kaduna, where the compiled list of pharmacies outnumbered the sample required. In the other 4 cities, a census of pharmacies was conducted. The pharmacy data were collected from 97 pharmacies in Ibadan, 96 pharmacies in Abuja, 89 pharmacies in Benin City, 80 pharmacies in Kaduna, 48 pharmacies in Ilorin, and 23 pharmacies in Zaria.

A sample of 100 PPMVs was randomly selected in each city from a compiled list of all PPMVs in Nigeria. However, in Abuja, where there were fewer PPMVs, all listed PPMVs were included in the survey sample. Ultimately, the PPMV data included 96 PPMVs in Zaria, 95 in Benin City, 94 in Abuja, and 90 each in Ibadan, Ilorin, and Kaduna. The total number successfully interviewed differs slightly from the intended 100 per city because some facilities on the list refused to participate in the survey or were closed.

In Kenya, the MLE baseline data collection combined both drug shops and pharmacies into one domain because we were not able to distinguish between drug shops and pharmacies for the purposes of this program evaluation. The Kenya baseline pharmacy/drug shop data were collected between August 2011 and October 2011 from a total of 223 pharmacies and drug shops across the 5 cities. In Mombasa and Nairobi, a random sample of 100 facilities was selected, whereas in Kakamega, Kisumu, and Machakos, a census of registered and operational pharmacies and drug shops was conducted. Ultimately, 62 pharmacies/drug shops completed interviews in Nairobi, 56 in Kisumu, 45 in Kakamega, 40 in Mombasa, and 30 in Machakos. Notably, the number of pharmacies/drug shops included in Mombasa and Nairobi was well below the 100 target because many of the smaller sites (i.e., drug shops) were not found, closed, or unavailable at the time of interview.

### Variables

The primary outcome of interest in this study was women's source of short-acting methods in selected urban areas of Nigeria and Kenya. As women who use these methods are likely different than women who use longer-acting methods or women who are non-users, we focused our attention on women using short-acting methods that can be supplied in pharmacies and drug shops. For descriptive purposes, we categorized women as short-acting method users versus long-acting method users, traditional method users, or non-users. Short-acting methods were defined as combined oral contraceptive pills, progestin-only contraceptive pills, male condoms, female condoms, injectables, emergency contraceptive pills, and spermicide. Long-acting methods included the implant, the intrauterine device (IUD), and the small number of women or their partners who were sterilized. Traditional method use included withdrawal, abstinence, and the Standard Days Method (SDM). Although SDM is generally considered a modern method, it was included as a traditional method in the baseline survey but was separated out in later rounds of data collection. For the purposes of this analysis, users of the lactational amenorrhea method (LAM) were coded as non-users (although it is also generally considered a modern method) since this is not a method that needs to be sourced from a facility.

The primary outcome of interest in this study was women's source of short-acting methods in selected urban areas of Nigeria and Kenya.

Women's sources of contraceptive methods were categorized as public facilities, private facilities, pharmacies, drug shops, and “other.” Public facilities included government hospitals, government health centers, government dispensaries, and other public-sector services. Private facilities included faith-based hospitals/clinics, private hospital/clinics, nursing/maternity homes, community health workers/traditional birth attendants, traditional healers, and other private facilities. “Other” sources included women who did not know where the method came from, such as women who acquired methods from husbands, boyfriends, fiancés, friends, or shops, or women who did not respond to the question. As mentioned earlier, pharmacies and drug shops were not distinguished in Kenya while in Nigeria, these are classified as 2 distinct groups with drug shops also being called PPMVs or chemists. For the multivariate analyses for Nigeria, we grouped the pharmacies and drug shops together, while for descriptive analyses these are kept separate. This was based on the descriptive similarities between drug shops and pharmacies in Nigeria and to facilitate comparison with the Kenya results presented.

The independent variables in this analysis included women's age, marital status, wealth, education, city of residence, religion, and whether or not she had any living children at the time of interview. As mentioned earlier, all women who had sex in the last year were included in the initial analysis of who was using a short-acting method; however, for the analysis of source of short-acting methods the sample was reduced to only women who reported a short-acting method as their current method.

### Analysis

We used descriptive statistics to examine the sample of women in selected urban areas of Nigeria and Kenya who have had sex in the last year by background demographic characteristics, contraceptive use patterns, and source of contraceptive method. On the supply side, we described a variety of outlet characteristics for pharmacies and drug shops in both countries as well as provision of family planning, staff training, and family planning prescription requirements.

We used multinomial regression to analyze factors related to women's choice of short-acting method source. All models controlled for demographic factors as outlined earlier. All women-level descriptive analyses were weighted using country-specific cross-city weights, and all multivariate analyses adjusted for clustering in the study design.

## RESULTS

In this section, we begin by describing the sample of women living in urban study areas in Nigeria and Kenya including their background demographic characteristics and their patterns of contraceptive use, which is particularly relevant to private-sector provision. Then we examine women's source of short-acting methods and factors associated with this choice.

### Women's Background Characteristics

Table 1 presents the background characteristics of the women who had sex in the last year in selected cities in Nigeria and Kenya. In both countries, a greater proportion of women were 25–34 years old compared with other age categories. The vast majority of women had some level of schooling (about 86% of the women in Nigeria and 94% in Kenya). Christianity was the predominant religion in Kenya (about 89%), whereas in Nigeria the distribution was split almost equally in half between Christians and Muslims. Most of the women who had sex in the last year in Nigeria (81%) and in Kenya (69%) were in union, and less than a quarter (15% in Nigeria and 22% in Kenya) had never been married. Similar proportions of women in Nigeria and Kenya (about 80%) had at least one living child. In Nigeria, the percentage distribution of women's city of residence ranged from 12% in Zaria to 24% in Kaduna. In contrast, Kenya had a greater weighted proportion of women from Nairobi (73%), with the lowest percentage coming from Machakos (1%).

**TABLE 1.** Descriptive Characteristics of Surveyed Women Who Had Sex in the Last Year in Selected Urban Areas, by Country, 2010/2011

Characteristic	Nigeria (N = 11,930)	Kenya (N = 7,085)
	No. (%)	No. (%)
Age category, years		
15–24	2429 (20.4)	2476 (35.0)
25–34	5296 (44.4)	3100 (43.8)
≥35	4205 (35.2)	1509 (21.3)
Highest education level		
None/Quranic	1653 (13.9)	429 (6.1)
Primary	2030 (17.0)	2673 (37.7)
Secondary	5140 (43.1)	2740 (38.7)
Higher	3027 (25.4)	1243 (17.5)
Missing	81 (0.7)	0 (0.0)
Religion		
Christian	5832 (48.9)	6318 (89.2)
Muslim	5969 (50.0)	621 (8.8)
No religion/other/none/missing	128 (1.1)	146 (2.1)
Marital/relationship status		
Never married	1835 (15.4)	1584 (22.4)
Married/living together	9703 (81.3)	4884 (68.9)
Divorced/separated	160 (1.3)	505 (7.1)
Widowed	93 (0.8)	108 (1.5)
Missing	139 (1.2)	4 (0.1)
Has at least one living child		
No	2353 (19.7)	1510 (21.3)
Yes	9577 (80.3)	5574 (78.7)
Wealth quintile		
Poorest	2206 (18.5)	1280 (18.1)
Poor	2434 (20.4)	1352 (19.1)
Middle	2510 (21.0)	1495 (21.1)
Rich	2462 (20.6)	1467 (20.7)
Richest	2319 (19.4)	1491 (21.1)
City (Nigeria/Kenya)		
Abuja/Nairobi	1594 (13.4)	5150 (72.7)
Benin City/Mombasa	1564 (13.1)	1325 (18.7)
Ibadan/Kisumu	2487 (20.8)	382 (5.4)
Ilorin/Machakos	1993 (16.7)	103 (1.4)
Kaduna/Kakamega	2853 (23.9)	126 (1.8)
Zaria/NA	1440 (12.1)	NA
Current contraceptive use		
No method	7409 (62.1)	3116 (44.0)
Long-acting method	406 (3.4)	508 (7.2)
Short-acting method	2750 (23.1)	3048 (43.0)
Natural/traditional methods	1365 (11.4)	413 (5.8)
Type of short-acting method among short-acting method users		
Injectable	722 (26.2)	1511 (49.6)
Oral contraceptive pill	372 (13.5)	860 (28.2)
Emergency contraception	214 (7.8)	87 (2.9)
Condom	1440 (52.4)	589 (19.3)
Spermicide	3 (0.1)	0 (0.0)

Note: All analyses are weighted; unweighted total for Nigeria was 11,873, and for Kenya 7,226.

### Contraceptive Use

Current use of a contraceptive method among women who had sex in the last year is shown at the bottom of Table 1. At baseline, 23% of women in Nigeria and 43% of women in Kenya were using a short-acting method. Use of natural or traditional methods was higher in Nigeria at 11%, compared with 6% in Kenya. Among short-acting method users in Nigeria, the majority (52%) were using the condom followed by injectables (26%) and the oral contraceptive pill (about 14%). In Kenya, the most commonly used short-acting method was the injectable (50%) followed by the oral contraceptive pill (28%) and then condoms (19%). The method distinction is relevant because, as mentioned earlier, drug shops and pharmacies have more regulatory barriers to the provision of injectables than other short-acting methods.

The most commonly used short-acting methods in both Nigeria and Kenya were condoms, injectables, and pills.

### Women's Method Source

Table 2 presents a description of where women obtained their short-acting methods at the time of the baseline survey. In Nigeria, drug shops were the major source of short-acting methods including oral contraceptive pills, emergency contraceptives, and condoms. In Kenya, pharmacies/drug shops were the most common single source of these same short-acting methods. The majority of injectable users obtained their method from public facilities in both countries, with only about 14% of women in Nigeria and 6% in Kenya obtaining injectables from pharmacies or drug shops. Nearly 8% of women in Nigeria did not know the source of their method; this likely reflects the husband/partner obtaining condoms. Although results are not shown here, the MLE survey did collect data from men on source of contraceptive method and, similar to women, men reported that the majority of condoms were obtained from a pharmacy or drug shop.

In both Nigeria and Kenya, drug shops or pharmacies were the most common source of short-acting contraceptive methods.

**TABLE 2.** Method Source Among Surveyed Women Using a Short-Acting Method and Who Had Sex in the Last Year in Selected Urban Areas, by Country and Method, 2010/2011

Source	Nigeria						Kenya				
	Injectable (n = 722)	OCP (n = 372)	EC (n = 214)	Condom (n = 1440)	Other modern (n = 3)	Total short-acting (N = 2750)	Injectable (n = 1511)	OCP (n = 860)	EC (n = 87)	Condom (n = 587)	Total short-acting (N = 3048)
Public facility	60.5	20.9	2.9	4.6	68.7	21.4	50.8	30.6	10.4	9.2	35.9
Private facility	22.3	4.2	3.3	2.4	31.3	8.0	42.7	25.5	9.0	10.1	30.6
Pharmacy (Nigeria); Pharmacy/drug shop (Kenya)	3.4	22.3	32.6	27.6	0.0	20.9	6.2	43.7	76.5	75.0	32.1
Drug shop/PPMV (Nigeria)	10.6	49.0	52.4	46.4	0.0	37.8	NA	NA	NA	NA	NA
Other	0.6	0.3	1.0	7.5	0.0	4.4	0.0	0.2	0.0	1.4	0.4
Don't know/missing	2.6	3.2	7.8	11.5	0.0	7.8	0.3	0.0	4.1	4.2	1.1
Total	100.0	100.0	100.0	100.0	100.0	100.0	100.0	100.0	100.0	100.0	100.0

Abbreviations: EC, emergency contraception; OCP, oral contraceptive pill; PPMV, proprietary patent medicine vendor.

### Drug Shop and Pharmacy Characteristics

Characteristics of drug shops and pharmacies in selected cities in Nigeria and Kenya are presented in Table 3. In Nigeria, many characteristics of pharmacies and drug shops were similar, including operating times and days of the week, proportion of interviewed staff that have ever received family planning training, and distribution of number of years in operation; drug shops tended to be a bit newer than pharmacies with 32% less than 5 years old compared with 20% of pharmacies. The vast majority of both pharmacies and drug shops in Nigeria offered family planning (96% and 87%, respectively) but less drug shops had family planning promotional materials on display at the time of interview (33% of pharmacies and 20% of drug shops).

**TABLE 3.** Characteristics of Drug Shops and Pharmacies Surveyed in Selected Urban Areas of Nigeria and Kenya, 2010/2011

	% of Nigerian Pharmacies (n = 433)	% of Nigerian Drug Shops (n = 555)	% of Kenyan Pharmacies/Drug Shops (n = 223)
Number of years open/in operation			
Less than 5	19.6	31.9	42.2
5 to 10	31.2	34.8	21.1
11 to 15	15.9	14.8	15.7
More than 15	16.9	12.3	11.7
Don't know	16.2	6.1	9.4
Missing	0.2	0.2	0.0
Number of operating hours per day			
Less than 5	0.2	0.5	1.4
5 to 10	13.2	16.6	26.5
11 to 15	73.2	72.4	66.8
More than 15	13.4	9.9	4.0
Missing	0.0	0.5	1.4
Number of operating days per week			
5	1.2	0.7	2.7
6	47.1	41.8	45.7
7	51.7	57.3	49.8
Missing	0.0	0.2	1.8
Number of regular, permanent staff			
Less than 5	69.8	92.4	79.8
5 to 10	22.6	1.6	11.7
11 to 15	1.9	0.0	5.4
More than 15	3.0	0.0	1.8
Missing	2.8	6.0	1.4
Outlet provides family planning methods			
Yes	95.8	87.0	98.2
No	3.0	12.6	1.8
Missing/Don't know	1.2	0.4	0.0
Observed family planning promotional materials on display			
Displayed	32.8	20.4	48.9
Not displayed	65.1	78.7	50.7
Missing	2.1	0.9	0.5
Person interviewed ever received training on family planning			
Yes	40.7	40.7	57.0
No	59.1	57.7	42.6
Don't know	0.2	1.6	0.0
Missing	0.0	0.0	0.5

In Kenya, the pharmacies and drug shops were relatively newly established, with more than 40% in operation for less than 5 years. As in Nigeria, almost all of the Kenyan outlets were open 6 or 7 days a week and about 67% were open 11–15 hours per day. The majority (about 80%) of pharmacies and drug shops in Kenya were small with less than 5 employees, and almost all (98%) provided family planning methods. A greater proportion (about half) of outlets in Kenya had family planning promotional materials on display at the time of audit, and 57% of those interviewed had received family planning training compared with slightly lower rates (41%) in Nigeria.

### Drug Shop and Pharmacy Provision of Family Planning

Pharmacies and drug shops that usually sell contraceptive methods were asked to list the modern methods they routinely provide in their shops. Male condoms were the most widely available method in Nigeria (>98%), whereas combined oral pills, condoms, and emergency contraception were all widely available (>90%) in Kenya (Table 4). Close to 70% of pharmacies in Nigeria and pharmacies and drug shops in Kenya offered injectables while only 20% of drug shops in Nigeria offered this method. Emergency contraceptives and progestin-only pills were more widely available in Kenya than in Nigeria; in contrast, a greater percentage of facilities (pharmacies at 32% and drugs shops at 14%) in Nigeria offered female condoms compared with Kenya (6%).

**TABLE 4.** Contraceptive Methods Offered in Surveyed Pharmacies and Drug Shops in Selected Urban Areas, by Facility Type and Country, 2010/2011

Method Offered	% of Nigerian Pharmacies (n = 415)	% of Nigerian Drug Shops (n = 483)	% of Kenyan Pharmacies/Drug Shops (n = 219)
Injectable	69.6	20.1	68.0
Combined oral pill	76.6	64.8	98.2
Progestin-only pill	4.3	1.9	14.6
Emergency contraception	70.4	37.9	92.7
Male condom	98.8	98.1	93.6
Female condom	31.8	14.1	5.9
Spermicide	1.5	0.4	NA

Table 5 features information on prescription requirements by method and type of outlet. In Nigeria, regardless of facility type, 39% to 56% of interviewed staff reported requiring a prescription for injectables, progestin-only pills, or combined oral pills. While condoms were the least restricted method in both countries, 14% of pharmacy respondents and 12% of drug shops in Nigeria reported that a prescription was required to offer the male condom. In Kenya, 56% required a prescription for injectables, 34% required a prescription for progestin-only pills, and 25% for combined oral pills.

Many pharmacies and drug shops in Nigeria and Kenya require prescriptions for injectables or pills, and some in Nigeria require them even for condoms.

**TABLE 5.** Surveyed Facilities Requiring a Prescription for Clients to Receive a Contraceptive Method in Selected Urban Areas, by Facility Type and Country, 2010/2011

	Nigerian Pharmacies	Nigerian Drug Shops	Kenyan Pharmacies/Drug Shops
	No. Providing Method (% Requiring Rx)	No. Providing Method (% Requiring Rx)	No. Providing Method (% Requiring Rx)
Injectable	289 (41.9)	97 (55.7)	149 (56.4)
Combined oral pill	318 (39.3)	313 (39.0)	215 (25.1)
Progestin only pill	18 (55.6)	9 (44.4)	32 (34.4)
Emergency contraceptives	292 (33.2)	183 (29.5)	203 (10.8)
Male condom	410 (14.2)	474 (12.2)	205 (1.0)
Female condom	132 (14.4)	68 (5.9)	13 (0.0)
Spermicide	6 (33.3)	2 (100.0)	NA

Abbreviation: Rx, prescription.

### Factors Associated With Where Women Obtained Short-Acting Methods

Focusing now on users of short-acting methods who had sex in the last year, Table 6 and Table 7 present multinomial logistic regression results comparing sources of these methods in Nigeria and Kenya. The small number of women who did not know or did not respond to the question of where they obtained their method were included as a separate category in the model but are not shown in the comparisons presented.

**TABLE 6.** Multinomial Logistic Regression Coefficients (Standard Errors) Comparing Source of Method Among Surveyed Women Using Short-Acting Methods, Selected Urban Areas of Nigeria, 2010/2011 (N=2,565)

	Pharmacy/Drug Shop vs. Public	Pharmacy/Drug Shop vs. Private	Public vs. Private
	*Column 1*	*Column 2*	*Column 3*
Marital status (Ref: Married)			
Never married	1.00 (0.41)*	0.10 (0.46)	−0.89 (0.57)
Divorced/separated/widowed	0.77 (0.41)^+^	−0.27 (0.45)	−1.04 (0.56)^+^
Religion (Ref: Christian)			
Muslim	−0.33 (0.13)**	−0.11 (0.21)	0.22 (0.22)
No religion	0.54 (0.80)	−1.31 (0.67)	−1.85 (0.91)*
Age (Ref: Age ≥35)			
<25	1.13 (0.21)***	0.54 (0.31)^+^	−0.59 (0.34)^+^
25–34	0.66 (0.12)***	0.45 (0.18)*	−0.21 (0.18)
Education (Ref: None)			
Primary	0.06 (0.26)	0.33 (0.38)	0.27 (0.38)
Secondary	0.26 (0.27)	0.12 (0.38)	−0.14 (0.38)
Higher than secondary	0.45 (0.28)	0.49 (0.41)	0.04 (0.42)
Wealth (Ref: Poorest)			
Poor	0.31 (0.19)	0.19 (0.28)	−0.11 (0.30)
Middle	0.26 (0.19)	0.14 (0.29)	−0.12 (0.30)
Rich	0.24 (0.20)	0.11 (0.31)	−0.13 (0.31)
Richest	0.42 (0.23)	−0.38 (0.31)	−0.80 (0.35)*
City (Ref: Ibadan)			
Abuja	−1.05 (0.19)***	0.31 (0.34)	1.37 (0.36)***
Benin City	−0.15 (0.20)	−0.43 (0.27)	−0.27 (0.31)
Ilorin	0.13 (0.16)	−0.62 (0.26)*	−0.75 (0.27)**
Kaduna	−0.86 (0.18)***	−0.73 (0.28)**	0.13 (0.29)
Zaria	−1.35 (0.24)***	−1.32 (0.39)***	0.03 (0.32)
Any living children	−1.22 (0.43)**	−1.48 (0.52)**	−0.26 (0.65)
Constant	1.30 (0.56)*	2.98 (0.68)***	1.69 (0.81)*

Note: Don't know, other, and missing options modeled but not shown in comparisons.

+ *P*≤.10;

* *P*≤.05;

** *P*≤.01;

*** *P*≤.001.

**TABLE 7.** Multinomial Logistic Regression Coefficients (Standard Errors) Comparing Source of Method Among Surveyed Women Using Short-Acting Methods, Selected Urban Areas of Kenya, 2010/2011 (N=3,049)

	Pharmacy/Drug Shop vs. Public	Pharmacy/Drug Shop vs. Private	Public vs. Private
	*Column 1*	*Column 2*	*Column 3*
Marital status (Ref: Married)			
Never married	1.17 (0.15)***	1.13 (0.19)***	−0.04 (0.17)
Divorced/separated/widowed	0.93 (0.18)***	0.66 (0.21)**	−0.26 (0.18)
Religion (Ref: Christian)			
Muslim	0.32 (0.23)	0.39 (0.25)	0.08 (0.22)
No religion	0.19 (0.48)	0.21 (0.46)	0.02 (0.47)
Age (Ref: Age ≥35)			
<25	−0.26 (0.16)	−0.17 (0.17)	0.09 (0.15)
25–34	−0.20 (0.14)	−0.17 (0.16)	0.02 (0.13)
Education (Ref: None)			
Primary	0.02 (0.30)	0.29 (0.31)	0.27 (0.23)
Secondary	0.22 (0.30)	0.52 (0.31)^+^	0.30 (0.22)
Higher than secondary	0.82 (0.32)*	0.74 (0.34)*	−0.08 (0.27)
Wealth (Ref: Poorest)			
Poor	0.24 (0.16)	0.13 (0.18)	−0.10 (0.14)
Middle	0.35 (0.17)*	0.12 (0.18)	−0.23 (0.15)
Rich	0.50 (0.16)**	0.27 (0.19)	−0.23 (0.17)
Richest	1.24 (0.20)***	0.26 (0.22)	−0.98 (0.20)***
City (Ref: Kisumu)			
Nairobi	0.73 (0.17)***	−0.17 (0.18)	−0.91 (0.15)***
Mombasa	0.63 (0.19)**	−0.67 (0.21)**	−1.30 (0.18)***
Machakos	0.13 (0.17)	0.70 (0.22)**	0.57 (0.20)**
Kakamega	0.73 (0.19)***	0.87 (0.24)***	1.60 (0.22)***
Any living children	−1.76 (0.19)***	−1.25 (0.21)***	0.51 (0.23)*
Constant	−0.28 (0.38)	0.22 (0.43)	0.50 (0.36)

Note: Don't know, other, and missing option modeled but not shown in comparisons.

+ *P*≤.10;

* *P*≤.05;

** *P*≤.01;

*** *P*≤.001.

Multiple comparisons are shown in both tables: drug shops/pharmacies compared with public facilities (Column 1); drug shops/pharmacies compared with private facilities (Column 2); and public vs. private facilities (Column 3). In both Nigeria and Kenya, unmarried women and women with no living children were significantly more likely to get their short-acting methods from a drug shop or pharmacy than from the public sector (and in Kenya, private facilities as well) compared with women in union or those with any children.

In Nigeria and Kenya, unmarried women and women with no living children were significantly more likely to get their short-acting methods from a drug shop or pharmacy than from the public sector compared with women in union or those with children.

Religion and age were important factors for source of short-acting methods in Nigeria but not in Kenya. Muslims in Nigeria were less likely than Christians to get their method from a drug shop/pharmacy than the public sector, and younger women were more likely than older women to get their short-acting method from drug shops or pharmacies in Nigeria compared with any other source. Education levels did not seem to make a significant difference in Nigeria, while in Kenya the most educated women were more likely than less educated women to get their method from a pharmacy/drug shop than elsewhere. Wealth status mattered in Kenya: the higher the wealth group of a woman, the more likely she was to purchase her method at a drug shop or pharmacy than a public-sector facility. There was no wealth effect observed in Nigeria.

Lastly, there were city-level differences in source of short-acting methods in each country. In Nigeria, women in the Northern cities—Abuja, Kaduna, and Zaria—were less likely to obtain their methods from a drug shop or pharmacy than from the public sector compared with women from Southern Ibadan city. In Kenya, women living in all of the cities (except Machakos) were more likely to get their short-acting method from a drug shop/pharmacy than the public sector compared with Kisumu.

## DISCUSSION

Drug shops and pharmacies offer an important and under-leveraged mechanism for expanding family planning access to women in urban Nigeria and Kenya, and potentially elsewhere, to help meet FP2020 goals. Vulnerable and harder-to-reach groups, such as younger, unmarried women and women who do not yet have children, are the most likely to benefit from increased access to family planning at drug shops and pharmacies.

Drug shops and pharmacies offer an important and under-leveraged mechanism for expanding family planning access to women in urban Nigeria and Kenya.

The results of this article show the crucial role drug shops and pharmacies play in the provision of short-acting methods to women in urban areas of Nigeria and Kenya. Pharmacies and drug shops are the dominant source for most short-acting family planning methods including pills, emergency contraception, and condoms for women in Nigeria and Kenya. While public facilities remain the main source for injectables, 6% of the women in Kenya and 14% in Nigeria obtain injectables from pharmacies and drug shops. Literature shows that regardless of the regulatory environment and the training level of staff, drug shops in Africa are providing injectable contraception.^13^ Major policy shifts in recent years toward expanded access to certain forms of family planning (such as implants and injectables) down to a community level, as well as successful trials of Sayana Press in multiple countries, indicate that drug shops and pharmacies may play an increasingly important role in the provision of new methods for certain populations.^9^^,^^14^^,^^17^ For instance, in 2012, Nigeria approved a policy to allow trained community health extension workers (CHEWs) to administer injectables and implants. This task shifting down to lower-level trained providers also presents an opportunity to continue to expand access points for injectables to drug shops and pharmacies in both Nigeria and Kenya.^9^^,^^14^^,^^19^

While public facilities remain the main source for injectables, a sizeable portion of women in Kenya and Nigeria obtain injectables from pharmacies and drug shops.

This study also shows that pharmacies and drug shops in both Nigeria and Kenya were highly accessible to women in terms of their hours and days open. Further, since these facilities sell a variety of products in addition to contraceptives, young and unmarried women (and men) may feel increased comfort levels with obtaining their contraceptive method at these sites.

While drug shops and pharmacies represent the dominant source of certain contraceptive methods in both Nigeria and Kenya, there are some significant limitations to these outlets in terms of family planning-related promotion and training that could be addressed. The relatively low prevalence of pharmacies and drug shops that had family planning promotional materials on display in Nigeria (less than a third) and in Kenya (in about half) indicates a potential missed opportunity for increased education and promotion of methods. In addition, the low levels of reported family planning-related training by interviewed staff in these outlets is a significant area for possible program expansion and improvement.

Many pharmacies and drug shops in Nigeria and Kenya did not have family planning promotional materials on display—potentially missing opportunities to improve education and promotion of contraceptive methods.

Taking advantage of expanding access to family planning products and services through urban drug shops and pharmacies may be best optimized by understanding who is likely to benefit the most. This study shows that drug shops and pharmacies are the preferred choice for obtaining short-acting methods among younger women and women without children in both countries. These findings have significant program and policy implications when targeting particular profiles of users to increase access to family planning and are consistent with earlier studies that show that drug shops and pharmacies are more accessible to marginalized populations.^9^^,^^11^^,^^12^^,^^15^ We also show sociodemographic differences between women getting short-acting methods from public and private health facilities versus pharmacies and drug shops in both countries. In Kenya, more educated and wealthy women are already more likely to access short-acting methods through pharmacies and drug shops. Kenya also has overall much higher rates of modern contraceptive use and a more robustly integrated family planning program in public-sector facilities compared with Nigeria. This system in Kenya may better service women who have already been pregnant or had a birth while there is still an opportunity to expand access to family planning for youth and women who have not yet had a birth through drug shops and pharmacies.

In Nigeria, we see lower levels of modern contraceptive use than in Kenya and a much less integrated set of family planning services in public-sector facilities. That being said, particularly in the South of the country, there is an incredibly active informal private sector for health services (including drug shops and pharmacies) that provides a unique opportunity to expand access to family planning methods to women who are less likely to visit a public health facility. This strategy may not be as appropriate (or is still more nascent) in the North of the country where women are more likely to obtain family planning services and most other health services through the public sector. This regional difference corresponds to existing and planned large-scale family planning program strategies that have been tailored to regional and cultural contexts through increased public sector and mobile outreach programs in the North and more private-sector access and programming in the South.^19^

It is worth noting that the distinctions found in women who choose drug shops over pharmacies may be related to choice of method. This may reflect that younger and unmarried women are using the more temporary methods (condoms, oral contraceptive pills, and emergency contraception) that are more accessible in these outlets, whereas older women who already have children are choosing more effective, longer-term methods, which are predominately available from a health facility (public or private). Further, women with any children may be more likely to get their short-acting method from a health facility if they are obtaining their method at the time of visiting a facility for a child health visit or some other service.

### Limitations

This study is not without limitations. First, this is a descriptive study and we cannot determine causality with the data available. Second, given that there are different method mixes across the countries, some of the distinctions found by source relate to method choice and not just to source of the method. Third, in the Kenya context, it was not possible to distinguish drug shops from pharmacies in the data collected. Fourth, the measure of a facility's prescription requirement for a given method is likely subject to social desirability bias given that the survey was interviewer administered.

## CONCLUSION

Pharmacies and drug shops have an important role to play in urban areas in support of attaining FP2020 national goals, especially in serving women with unmet need for short-acting methods. Ensuring access to a full array of methods at drug shops and pharmacies should be an important goal of each of the FP2020 country implementation plans moving forward. These accessible outlets embedded in urban communities in Nigeria and Kenya and elsewhere provide a rich opportunity to further increase access to much-needed contraceptive methods.

## Notes

**Peer Reviewed**
